# Supplementary material for: Cluster Analysis in Diabetes Research: A Systematic Review Enhanced by a Cross-Sectional Study
Source: J Clin Med. 2025 May 21;14(10):3588. doi: 10.3390/jcm14103588 (PMC12112067; doi:10.3390/jcm14103588)
Supplement: Supplementary file 1 [file jcm-14-03588-s001.zip › Supplementary Materials_Tables S1-S8.pdf]

Table S1. Characteristics of selected studies.

N	First author, publication year	Geographic region	Study design	Data source	Sample size and its characteristics	Diabetes diagnostic criteria
1	Wang et al., 2021 [12]	China (Asia)	Cross-sectional study	Patients admitted to a tertiary care center, January 2017 - December 2018	1,152 inpatients aged ≥ 18 years at the time of first diagnosis of diabetes with the proportion of T2D, T1D and LADA of 95.45%, 2.54% and 2.01%, respectively	WHO, 1999
2	Herder et al., 2021 [27]	Germany (Other region)	Cross-sectional analysis based on data from a prospective cohort study	Data from the German Diabetes Study (GDS), prospective observational cohort study, September 2005 - December 2011	414 individuals with recent-onset diabetes (known diabetes duration ≤ 1 year)	ADA, 2018
3	Xiong et al., 2021 [13]	China (Asia)	Retrospective cohort study	Retrospective cohort of patients from the National Clinical Research Center for Metabolic Diseases Diabetes Center, including the Department of Endocrinology and Nephrology of the Second Xiangya Hospital of Central South University, Changsha, China, January 2010 - November 2018.	Of the 5,414 patients aged 18 years or older at the onset of T2D, with an average disease duration of 8.6 ± 6.3 years (57% men), 5,011 participants were included in the cluster analysis.	FPG ≥7.0 mmol/L (126 mg/dL); random plasma glucose ≥11.1 mmol/L (200 mg/dL); or 2hPG ≥11.1 mmol/L; or HbA1c ≥ 6.5%.
4	Song et al., 2022 [14]	China (Asia)	Cross-sectional study	Department of Endocrinology, Zhongda Hospital, Southeast University; Affiliated Jiangning Hospital of Nanjing Medical University; The Second People's Hospital of Wuhu; Nanjing Central Hospital; Xigang Community Health Service Center, July 2018 - July 2021	1,017 inpatients with new-onset diabetes with a disease duration of less than two years	WHO, 1999
5	Zhang et al., 2022 [15]	China (Asia)	Retrospective cohort study	Patients with diabetes who were hospitalized in the Endocrinology Department of the First Affiliated Hospital of Nanchang University between December 2020 and March 2022, and whose blood glucose levels met the standard at discharge. All medical information was obtained from electronic health records.	1,332 participants. Seven (0.5%) of the participants presented with T1D, 44 (3.3%) had LADA, and 1,281 (96.2%) had T2D.	FPG ≥7.0 mmol/L, 2hPG ≥ 11.1 mmol/L, and/or HbA1c ≥ 6.5%
6	Pigeyre et al., 2022 [59]	578 clinical sites in 40 countries	Secondary analysis of RCT data	Outcome Reduction with Initial Glargine Intervention (ORIGIN) trial, 2003-2005. Participants were monitored for cardiovascular and renal events for a median follow-up of 6.2 years.	7,017 participants aged ≥ 50 years, of whom 6,492 had established diabetes and 525 had newly diagnosed diabetes.	FPG ≥ 126 mg/dL or a PPG ≥ 200 mg/dL, or a previous diagnosis of diabetes
7	Tanabe et al., 2022 [28]	Japan (Asia)	Three-year prospective cohort study	Fukushima Diabetes, Endocrinology, and Metabolism cohort, January 2018 - February 2020. The median follow-up period was 805 days (388-1113).	586 Japanese participants with either T1D or T2D	FPG ≥ 126 mg/dL, casual plasma glucose ≥ 200 mg/dL, HbA1c ≥ 6.5%, or if the

						participant regularly used antihyperglycemic drugs
**8**	Saito et al., 2022 [29]	Japan (Asia)	Retrospective cohort study	Electronic health records of patients who underwent the educational admission program at the Center for Diabetes, Endocrinology and Metabolism, Shizuoka Prefectural Shizuoka General Hospital, 2009 – 2020. Longitudinal changes in HbA1c were assessed (n = 651, median follow up: 2.0 years (IQR 0.4–4.2).	651 participants with a median age of 63 years (51-72). Women accounted for 36%. The number of participants with T1D was 32 (4.9%), T2D - 546 (83.9%), LADA - 73 (11.2%).	ICD, 10the revision: E10 and E11
**9**	Danquah et al., 2023 [37]	Ghana (Other region)	Cross-sectional study	Multi-center, cross-sectional study titled Research on Obesity and Diabetes among African Migrants (RODAM), July 2012 - September 2015	541 Ghanaians with adult-onset DM and a mean age of 53.2 ± 9.5 years, ranged from 25 to 70 years; 44% were men. The median duration of DM was 5 years (1–11). Most individuals lived in Amsterdam (32%), followed by urban Ghana (25%), London (19%), Berlin (13%) and rural Ghana (10%). The mean HbA1c was 7.7 ± 2.2%. More than two-thirds of individuals were overweight or obese (BMI ≥ 25 kg/m^2) and 56% had abdominal obesity.	FPG ≥ 7.0 mmol/L, documented use of glucose-lowering medication or self-reported diabetes.
**10**	Lu et al., 2024 [33]	USA (Other region)	Retrospective cohort study	Electronic health records, Kirklin Clinic of the University of Alabama at Birmingham (UAB), serving as an ambulatory referral center, January 2010 - December 2019. Measurements closest to the date of first diabetes diagnosis were used.	1,194 participants diagnosed with diabetes between January 2010 and December 2019. Overall, the UAB adult diabetes patient cohort had a mean age of 54.8 years (CI, 54.1-55.5), an HbA1c of 8.7% (CI, 8.6-8.9), and a BMI of 33 kg/m^2 (CI, 32.6-33.4).	ICD-9 clinical Modification: 249 and 250;<br>ICD-10 clinical Modification: E08, E09, E10, E11, and E13
**11**	Anjana et al., 2020 [30]	India (Asia)	Retrospective cohort study	Electronic health records from a network of 50 diabetes centers across nine states of India; Replication was performed using a dataset derived from the Indian Council of Medical Research-India Diabetes (ICMR-INDIAB) study.	19,084 individuals with T2D with a disease duration of less than five years and aged 10–97 years were included for primary clustering; A total of 2,204 participants with T2D were involved for validation. GADA were measured in a selected subset of patients.	FPG ≥126mg/dL (7 mmol/L) and/or 2hPG ≥200mg/dL (11.1mmol/L) and/or if the patient has been prescribed pharmacotherapy for diabetes. T2D was diagnosed in the absence of ketosis, good β-cell reserve indicated by FCP > 0.6 pmol/mL, no presence of calculi on abdominal radiograph, and response to oral hypoglycemic medications for at least 2 years.

**12**	Huang et al., 2021 [16]	China (Asia)	Cross-sectional study	Tongji Hospital, March 2016 - June 2019	246 T2D patients. All patients had a history of taking antidiabetic drugs for >3 months.	ADA, 2010 (FG ≥ 7.0 mmol/L, random glucose ≥ 11.1 mmol/L, or the positive results after an oral glucose tolerance test)
**13**	Xing et al., 2021 [17]	China (Asia)	Cross-sectional study	Medical records from No.1 Shenzhen People's Hospital, January 2018 - November 2019	1,060 T2D participants with a median age of 57 years (IQR 50–65), 61% were men. Newly diagnosed diabetes was present in 16% of patients, and 84% of patients had long-term diabetes.	WHO, 1999
**14**	Gao et al., 2022 [18]	China (Asia)	Nationwide population-based cohort study	Data from the China Health and Nutrition Survey (CHNS), a nationwide cohort, 1989–2009. The observation period for retrospective trajectories began in 2009 (year 0).	557 participants aged ≥ 18 years with newly diagnosed T2D. GADA were unavailable in the CHNS survey.	ADA, 2020 (FBG ≥ 7.0 mmol/L (126 mg/ dl) or HbA1c ≥6.5% (48 mmol/mol)
**15**	Christensen et al., 2022 [38]	Denmark (Other region)	Nationwide population-based cohort study	Danish Centre for Strategic Research in Type 2 Diabetes (DD2) cohort. Enrollment took place in hospital specialist outpatient clinics or general practitioners' offices, November 2010 - February 2015	3,529 individuals aged ≥18 years with new clinically diagnosed T2D with or without initiation of glucose-lowering therapy. The mean age at diagnosis of diabetes was 59.2 ± 11.2 years. Fifty-nine percent were men. GADA positive patients were excluded.	WHO criteria, before 2012 based on OGTT and after 2012 based on HbA1c >48 mmol/mol (6.5%).
**16**	Peng et al., 2022 [19]	China (Asia)	Retrospective cohort study	Inpatients with T2D from Tongji Hospital, 2017-2019	285 inpatients with T2D. T2D patients were newly-diagnosed through the classical oral glucose tolerance test after admission or confirmed according to the medical history. GADA were not measured.	FG ≥ 7.0 mmol/L, random glucose ≥11.1 mmol/L, or 2hPG ≥11.1mmol/L
**17**	Zou et al., 2022 [58]	Multiple countries	Secondary analysis of RCT data	Five randomised, double-blinded clinical trials of canagliflozin.	6,365 participants with T2D. GADA data were missing. The mean age was 58.1 ± 9.4 years; 57% were males.	-
**18**	Choi et al., 2022 [34]	USA (Other region)	Cross-sectional study	Data from ten continuous waves of the National Health and Nutrition Examination Surveys (NHANES), 1999–2018	825 US women with a median age of 58.5 years; the percentage of non-Hispanic whites, non-Hispanic blacks, and Hispanics was 61.2%, 19.0%, and 19.8%, respectively.	Self-reported
**19**	Wang et al., 2022 [39]	Singapore (Asia)	Prospective cohort study	Singapore Study of Macro-Angiopathy and Micro-Vascular Reactivity in Type 2 Diabetes (SMART2D). Patients were recruited from outpatient clinics of a secondary hospital and an adjacent primary care medical facility, 2011 - 2014. Participants were recalled for a research visit every 3 years, and also followed up by reviewing electronic health records. The median follow-up period was 7.3 years (IQR 6.7–7.7).	687 individuals with recent-onset T2D (diabetes duration ≤ 5 years). GADA were not measured.	T2D was diagnosed by a physician after ruling out T1D and diabetes attributable to specific causes.
**20**	Abdul-Ghani et al., 2022 [40]	USA, Qatar (Other region/Asia)	Secondary analysis of RCT data	EDICT, Efficacy and Durability of Initial Combination Therapy for Type 2 Diabetes, and Qatar studies	The EDICT study initially included 305 patients with T2D who were drug-naive and recently diagnosed (<2 years). Participants were middle-aged (age 46±1 years), obese (BMI 36.1 ± 0.4 kg/m^2), with a disease duration 4.9 ± 3.8 months, and were primarily Mexican-American (70%); Patients positive for GADA were excluded. Only 100 participants were included in the cluster analysis.	-

					The Qatar study initially involved 331 poorly controlled (HbA1c > 7.5%) T2D patients. Participants were middle-aged (age 52 ± 1 years), less obese (BMI 30.5 ± 0.3 kg/m^2), had longstanding diabetes (10.9 ± 0.3 years), worse glycemic control and were receiving metformin plus sulphonylurea; 70% were Arab and 30% were South Asian. Only 100 participants were included in the cluster analysis.	
**21**	Preechasuk et al., 2022 [41]	Thailand (Asia)	Prospective cohort study	Siriraj Diabetes Registry. The median follow-up period was 21.1 months (9.2–35.2).	721 participants with diagnosed T2D within 2 years from the registration date. The mean age was 53.4 ± 11.3 years, 58.9% were women.	-
**22**	Wang et al., 2022 [20]	China (Asia)	Prospective cohort study	Prospective cohort study of 10,375 adults aged ≥ 40 years from Jiading District, the suburb of Shanghai, China, March - August 2010. Follow-up examination was conducted between August 2014 and May 2015. Follow-up interviews were conducted with 688 participants out of a total of 1,130.	1,130 adults with newly-diagnosed T2D and a mean age of 61.4 ± 9.6 years; 41% were males. GADA were not measured.	Newly-diagnosed diabetes was defined by FPG ≥ 7.0 mmol/L (126 mg/dL) and/or 2hPG ≥ 11.1 mmol/L (200 mg/dL) and/or HbA1c ≥ 6.5%
**23**	Wang et al., 2023 [21]	China (Asia)	Secondary analysis of RCT data	Randomized controlled trials: MARCH, metformin and acarbose in Chinese patients as the initial hypoglycemic treatment, and CONFIDENCE, comparison of glycemic control and β-cell function among newly diagnosed patients with type 2 diabetes treated with exenatide, insulin or pioglitazone, cohorts	590 newly diagnosed T2D participants from the MARCH cohort; 392 newly diagnosed T2D participants from the CONFIDENCE cohort. Individuals who tested positive for GADA were excluded from the CONFIDENCE cohort.	WHO, 1999
**24**	Li et al., 2023 [42]	The Netherlands (Other region)	Prospective cohort study	Diabetes Care System (DCS), a dynamic prospective cohort of the natural course of T2D from 103 general practitioners in the West Friesland region of the Netherlands, 1998–2019. Five-year intensive intervention was analyzed.	2,935 individuals with newly diagnosed T2D. Participants were negative for GADA.	T2D was defined by the presence of at least one of the following: 1) one or more classic symptoms (excessive thirst, polyuria, weight loss, hunger, or pruritus) in combination with either FPG ≥7.0 mmol/L or random plasma glucose ≥11.1 mmol/L; 2) in the absence of the above symptoms - at least two elevated plasma glucose concentrations on two different occasions.
**25**	Hwang et al., 2023 [43]	Korea (Asia)	Community-based	Korean Genome and Epidemiology Study conducted by the Korean Center for Disease Control and Prevention. Baseline examinations	756 patients with newly diagnosed drug-naive T2D. GADA were not measured.	ADA, 2022

			prospective cohort study	took place in 2001–2002, and follow-up assessments were carried out every two years, with data up to 2015–2016 used in this study. The follow-up period was up to 14 years.		
**26**	Li et al., 2023 [22]	China (Asia)	Cross-sectional study	T2D patients admitted to the Endocrinology and Metabolism Department of Lanzhou University Second Hospital, May 2019 - August 2022	547 newly diagnosed T2D patients with the diabetes duration of less than one year. GADA data were missing.	WHO, 1999
**27**	Wang et al., 2024 [23]	China (Asia)	Secondary analysis of RCT data	MARCH, metformin and acarbose in Chinese patients as the initial hypoglycemic treatment	590 newly diagnosed T2D participants. At week 48, 346 participants completed follow-up.	WHO, 1999
**28**	Arora et al., 2024 [31]	India (Asia)	Cross-sectional study	Department of Endocrinology at a tertiary care hospital, New Delhi, August 2021 - June 2022	469 participants with newly diagnosed T2D or diabetes duration of less than two years (70%). The mean age at diagnosis was 47.2 ± 10.8 years, mean BMI was 26.16 ± 4.8 kg/m^2; 46.3% were males and 53.7% were females. A total of 49.2% were on treatment at the time of enrolment in the study. GADA positive individuals were excluded.	WHO (FPG >126 mg/dL (7.0 mmol/L) or 2hPG >200 mg/dL (11.1 mmol/L))
**29**	Li et al., 2024 [24]	China (Asia)	Cross-sectional study	From eight centers of the REACTION (Risk evaluation of cancers in Chinese diabetic individuals) study, March - December 2012	6,369 participants with newly diagnosed diabetes, mean age of 60.62 ± 9.11 years and mean BMI of 25.61 ± 3.58; 36.5% were men. GADA were not measured.	ADA, 2011 (FBG ≥7.0 mmol/L or PBG ≥11.1 mmol/L or HbA1c ≥6.5%)
**30**	Tripathi et al., 2024 [32]	India (Asia)	Cohort Study on a 1-Year Online Intensive Lifestyle Intervention for T2D Management	Data from the Freedom from diabetes clinic running an online diabetes management program. Data collected from patients enrolled between 2013 and 2023	281 patients with T2D duration of less than two years. Among the study population, 65.6% were using glucose-lowering medications, 27% were drug-naive, and 7.4% were on insulin in addition to oral hypoglycemic agents. The mean age was 42.3 ± 11.3 years, 59.4% were male.	WHO, 2019
**31**	Bayoumi et al., 2024 [44]	United Arab Emirates (Asia)	Cross-sectional study	Electronic health records of patients who underwent random screening between January 2020 and December 2022 at the outpatient departments of the Dubai Diabetes Centre and Dubai Hospital, Dubai, UAE	348 Emirati Arab T2D patients with a mean age of 56 ± 10.6 years and a mean diabetes duration of 14 ± 8.1 years. Fifty-two percent were women. The mean BMI was 31 ± 5.7 kg/m^2 and the mean age at diagnosis was 42 ± 10.7 years. Patients were tested for GADA.	-
**32**	Li et al., 2024 [45]	The Netherlands, Scotland (Other regions)	Retrospective cohort study	Hoorn Diabetes Care System (DCS, the Netherlands, median follow-up 11.2 years, 1998–2019) and Genetics of Diabetes Audit and Research in Tayside Scotland (GoDARTS, Scotland, median follow-up 12.3 years, 2003–2018).	9,199 T2D individuals with age at diagnosis ≥35 years and negative GADA.<br><br>The DCS cohort consisted of 3,054 individuals with a mean age of 63 years; 52.3% were men.<br><br>The GoDARTS cohort included 6,145 individuals with a mean age of 64 years; 53.3% were men.	In the DSC cohort, the diagnosis of T2D was defined by the presence of at least one of the following: 1) one or more classic symptoms (excessive thirst, polyuria, weight loss, hunger, or pruritus) in combination with either FPG ≥7.0 mmol/L or

						random plasma glucose ≥11.1 mmol/L; 2) in the absence of the above symptoms - at least two elevated plasma glucose concentrations on two different occasions
**33**	Lugner et al., 2021 [46]	Sweden (Other region)	Population-based cohort study	The National Diabetes Register of Sweden contains nationwide hospital discharge information, diagnoses and procedures from all specialist care (in-hospital and outpatient), data on ICD-10 codes, procedure codes and date of contact. The median follow-up time was 5.2 years (IQR 3.7-7.3).	114,231 participants with newly diagnosed T2D. The mean age was 62.8 ± 12.78 years and 43.1% of the individuals were women.	Diabetes in the Swedish National Patient Register is registered according to ICD 9th and 10th revision. T2D was defined as diabetes in patients ≥40 years at diabetes diagnosis, treated with diet and with or without oral glucose-lowering agents.
**34**	Cojic et al., 2021 [47]	Montenegro (Other region)	Cross-sectional study	From a cross-sectional study conducted at Primary Health Care Center (PHCC), Podgorica, Montenegro, May - June 2018.	95 patients with T2D and a mean age of 61.01 ± 7.94 years, who were treated with metformin. Fifty-three percent were males. The mean duration of diabetes was 6.01 ± 3.03 years.	ADA, 2011
**35**	Grimsmann et al., 2022 [49]	Germany, Austria (Other regions)	Multinational, population-based cohort study	German/Austrian Diabetes Follow-up Registry, 1995-2019. Follow-up data on HbA1c were obtained after a median diabetes duration of 1.8 years (IQR 1.4-2.1).	56,869 newly-diagnosed individuals. Thirteen percent were diagnosed with T1D, and 87% with T2D. The median age at diagnosis was 40 years (31- 48); 59% were men.	-
**36**	Manzini et al., 2022 [50]	Spain (Other region)	Retrospective cohort study	Electronic health records extracted from the Information System for the Development of Research in Primary Care (SIDIAP) database, January 2013 - December 2017	11,028 patients diagnosed with T2D. The mean age was 69.8 ± 10.6 years; 52% were men.	ICD, 10the revision: E11 and E14 and their subcodes
**37**	Wang et al., 2023 [25]	China (Asia)	Retrospective cohort study	Data from the Yidu Cloud data management platform on patients admitted to the Department of Endocrinology, the Second Affiliated Hospital of Dalian Medical University, January 2018 - October 2020	2,267 hospitalized patients with T2D and a median age of 63 years (56-70), including 54.3% males and 45.7% females.	WHO, 1999
**38**	Abbasi et al., 2023 [35]	USA (Other region)	Retrospective cohort study	Texas Children Hospital, July 2016 - July 2019. The mean follow-up duration was 2.7 years, with data analysis conducted retrospectively.	449 children and adolescents (< 19 years of age) with autoantibody-negative T2D were included in the cluster analysis. Among the 722 patients in the database, the median age at diagnosis was 13.7 years. Sixty-two percent were females, and 73.5% were obese. The racial/ethnic distribution was 58.3% Hispanic, 29.4% non- Hispanic Black, 9.2% non-Hispanic White, 3% Asian, and 0.1% other races.	-

**39**	Wang et al., 2023 [26]	China (Asia)	Cross-sectional study	Patients who underwent clinical therapy at the National Clinical Research Center for Metabolic Diseases, Second Xiangya Hospital of Central South University, January 2000 - December 2020.	1,410 T1D inpatients with a median age at diagnosis of 23 years (12-34.7), a median diabetes duration of 0.2 years (0-1.6), and a mean BMI of 19.5 ± 3.7 kg/m^2. Fifty-five percent were men.	T1D was defined when at least 2 of the following 3 diagnostic criteria were met: (1) age at onset between 6 months and 30 years, (2) insulin dependence from disease onset, and (3) DKA and/or DK at diagnosis. All patients with T1D were tested for islet autoantibodies to confirm the diagnosis.
**40**	Cojic et al., 2024 [48]	Montenegro (Other region)	Cross-sectional study	Primary Health Care Center Podgorica, Montenegro	424 T2D participants with a mean age of 66.19 ± 11.14 years and a mean disease duration of 8.67 ± 4.93 years; 49% were men.	ICD, 10the revision: E11 and E14
**41**	Somolinos-Simon et al., 2024 [36]	USA (Other region)	Retrospective cohort study	T1D Exchange Registry (T1DXR), containing data from 83 clinic sites in the United States. The database consisted of longitudinal information of individuals who received routine clinical care between 2007 and 2018. The number of patients with complete data decreased to 3,552 by five years after enrollment (Visit 5).	6,302 participants with T1D	Clinical diagnosis of T1D and either the presence of islet cell antibodies, or, if antibodies were negative or unknown, then insulin should have been started at or shortly after diagnosis and used continuously thereafter

Note. 2hPG, 2-h postprandial plasma glucose; ADA, American Diabetes Association; BMI, body mass index; DK, diabetic ketosis; DKA, diabetic ketoacidosis; DM, diabetes mellitus; FBG, fasting blood glucose; FCP, fasting C-peptide; FG, fasting glucose; FPG, fasting plasma glucose; GADA, glutamic acid decarboxylase antibodies; HbA1c, hemoglobin A1c; ICD, International Classification of Diseases; LADA, latent autoimmune diabetes in adults; OGTT, oral glucose tolerance test; PBG, postprandial blood glucose; RCT, randomized controlled trial; T1D, type 1 diabetes; T2D, type 2 diabetes; WHO, World Health Organization.

Table S2. Cluster analysis methodologies used in the selected studies.

N	First author, publication year	Clustering and dimensionality reduction methods	Methods for determining the number of clusters
1	Wang et al., 2021 [12]	K-means; T-distributed stochastic neighbour embedding visualization	Predetermined number of clusters
2	Herder et al., 2021 [27]	Nearest centroid clustering approach	Predetermined number of clusters
3	Xiong et al., 2021 [13]	Two-step clustering	Silhouette width method, Log-Likelihood Distance and Schwarz's Bayesian criterion
4	Song et al., 2022 [14]	Two-step clustering	Predetermined number of clusters
5	Zhang et al., 2022 [15]	Two-step clustering	Silhouette width method
6	Pigeyre et al., 2022 [59]	Nearest centroid clustering approach	Predetermined number of clusters
7	Tanabe et al., 2022 [28]	K-means	Silhouette width method
8	Saito et al., 2022 [29]	K-means	Predetermined number of clusters
9	Danquah et al., 2023 [37]	Two-step clustering	First approach: Silhouette width method. Second approach: Cluster size and Dendrogram of explained variance
10	Lu et al., 2024 [33]	Hierarchical clustering	Predetermined number of clusters
11	Anjana et al., 2020 [30]	K-means	Silhouette width method
12	Huang et al., 2021 [16]	K-means; T-distributed stochastic neighbour embedding visualization	Predetermined number of clusters
13	Xing et al., 2021 [17]	K-means	Elbow method
14	Gao et al., 2022 [18]	K-means	Predetermined number of clusters
15	Christensen et al., 2022 [38]	K-means	Replication of the Swedish T2D clusters: Predetermined number of clusters; New DD2 clusters: Silhouette width, elbow and gap statistic methods
16	Peng et al., 2022 [19]	K-means; T-distributed stochastic neighbour embedding visualization	Predetermined number of clusters
17	Zou et al., 2022 [58]	K-means and Hierarchical clustering	Predetermined number of clusters
18	Choi et al., 2022 [34]	K-means	Silhouette width and Elbow methods
19	Wang et al., 2022 [39]	K-means	NbClust package in R
20	Abdul-Ghani et al., 2022 [40]	K-means	Silhouette width method and Dendrogram analysis
21	Preechasuk et al., 2022 [41]	K-means	Silhouette width and Elbow methods
22	Wang et al., 2022 [20]	K-means	Predetermined number of clusters
23	Wang et al., 2023 [21]	K-means; T-distributed stochastic neighbour embedding visualization	Predetermined number of clusters
24	Li et al., 2023 [42]	K-means	Gap statistic

25	Hwang et al., 2023 [43]	K-means and Hierarchical clustering	Silhouette width method
26	Li et al., 2023 [22]	K-means	Predetermined number of clusters
27	Wang et al., 2024 [23]	K-means; T-distributed stochastic neighbour embedding visualization	Predetermined number of clusters
28	Arora et al., 2024 [31]	K-means	Silhouette width method
29	Li et al., 2024 [24]	K-means	Silhouette width method
30	Tripathi et al., 2024 [32]	K-means	Silhouette width and Elbow methods
31	Bayoumi et al., 2024 [44]	Hierarchical clustering, K-means and Kohonen algorithm	Two-step process, Silhouette Index and Kohonen algorithm
32	Li et al., 2024 [45]	K-means	Predetermined number of clusters
33	Lugner et al., 2021 [46]	K-means	Elbow, silhouette width, gap statistics, Hopkins statistic methods
34	Cojic et al., 2021 [47]	Agglomerative hierarchical clustering	Dendrogram analysis and Ward's method
35	Grimsmann et al., 2022 [49]	Agglomerative hierarchical clustering	Cubic clustering criterion, Pseudo-F statistic, Pseudo-t^2 statistic
36	Manzini et al., 2022 [50]	K-means, CLARA (clustering large applications), and Hierarchical clustering	Silhouette width method
37	Wang et al., 2023 [25]	K-means	Silhouette width method
38	Abbasi et al., 2023 [35]	K-prototype clustering	Silhouette width method
39	Wang et al., 2023 [26]	Two-step clustering	Log-Likelihood Distance and Schwarz's Bayesian criterion
40	Cojic et al., 2024 [48]	Two-step clustering	Silhouette width method and Dendrogram analysis
41	Somolinos-Simon et al., 2024 [36]	K-means	Average Silhouette and Elbow methods

Note. T2D, type 2 diabetes.

Table S3. Variables used in cluster analysis.

N	First author, publication year	Age at diagnosis/onset/manifestation/admission/baseline/first visit	BMI	HbA1c	HOMA2-IR/ HOMA-IR	HOMA2-B/ HOMA-B	GADA/GAD65Ab	GADA, ZnT8A, IA-2A, ICA-40KD, ICA-120KD, ICA-64KD, IAA-5.8KD	GADA, IA-2A, and ZnT8A	β-cell antibody status	TG	HDL-C	LDL-C	TC	TYG	non-HDL-C	FPG/ FBG	2hPG	Insulin	C-peptide, fasting or fasting and stimulated	ISI	UA	eGFR	mAlb	Creatinine and ACR	Vitamin D	Waist circumference	Systolic blood pressure	Diastolic blood pressure	Diabetic ketoacidosis	Hypertension/ dyslipidemia/ hyperuricemia	Age	Diabetes duration	Gender	Race/ethnicity	HLA status	Family history of T2D	CVD, CKD, neuropathy, high blood pressure, ophthalmological complications and antidiabetic drugs
1	Wang et al., 2021 [12]	+	+	+	+	+	+																															
2	Herder et al., 2021 [27]	+	+	+	+	+	+																															
3a	Xiong et al., 2021 [13]: Validation of the applicability of the European classification	+	+	+	+	+	+																															
3b	Xiong et al., 2021 [13]: Remodeling the cluster analysis	+	+	+	+	+	+				+											+																
4	Song et al., 2022 [14]	+	+	+	+	+		+																														
5	Zhang et al., 2022 [15]	+	+	+	+	+	+																															

6	Pigeyre et al., 2022 [59]	+	+	+			+													+																		
7	Tanabe et al., 2022 [28]	+	+	+	+	+	+																																
8	Saito et al., 2022 [29]	+	+	+	+	+	+																																
9a	Danquah et al., 2023 [37]: First approach	+	+	+	+	+	+																																
9b	Danquah et al., 2023 [37]: Second approach	+															+		+								+												
10	Lu et al., 2024 [33]	+	+	+	+	+	+																																
11	Anjana et al., 2020 [30]	+	+	+							+	+								+							+												
12	Huang et al., 2021 [16]	+	+	+	+	+																																	
13	Xing et al., 2021 [17]	+	+	+	+	+																																	
14	Gao et al., 2022 [18]	+	+	+	+	+																																	
15	Christensen et al., 2022 [38]	+	+	+	+	+																																	
16	Peng et al., 2022 [19]	+	+	+	+	+																																	
17	Zou et al., 2022 [58]		+	+	+	+																										+							
18	Choi et al., 2022 [34]	+	+	+	+	+																																	
19	Wang et al., 2022 [39]	+	+	+	+	+																																	
20	Abdul-Ghani et al., 2022 [40]	+	+	+	+	+																																	
21	Preechasuk et al., 2022 [41]	+	+	+							+	+																											
22	Wang et al., 2022 [20]	+	+	+	+	+											+	+																					
23	Wang et al., 2023 [21]	+	+	+	+	+																																	
24	Li et al., 2023 [42]	+	+	+								+								+																			
25	Hwang et al., 2023 [43]	+	+	+	+	+																																	

26	Li et al., 2023 [22]	+	+	+	+	+																																
27	Wang et al., 2024 [23]	+	+	+	+	+																																
28	Arora et al., 2024 [31]	+	+	+	+	+																					+											
29	Li et al., 2024 [24]	+	+	+	+	+																																
30	Tripathi et al., 2024 [32]	+	+	+	+	+																																
31	Bayoumi et al., 2024 [44]	+	+	+													+		+																			
32	Li et al., 2024 [45]	+	+	+								+								+																		
33	Lugner et al., 2021 [46]	+	+	+							+	+	+										+					+	+									
34	Cojic et al., 2021 [47]		+	+							+	+	+	+	+		+		+							+						+	+					
35	Grimsmann et al., 2022 [49]	+	+	+						+																				+	+			+				
36	Manzini et al., 2022 [50]	+	+	+							+	+	+	+											+			+	+			+	+	+				+
37	Wang et al., 2023 [25]		+		+						+			+			+		+		+	+		+				+	+									
38	Abbasi et al., 2023 [35]	+	+	+												+																		+	+			
39	Wang et al., 2023 [26]	+							+											+																+	+	
40	Cojic et al., 2024 [48]		+	+							+	+	+	+	+		+		+							+						+	+					
41	Somolinos-Simon et al., 2024 [36]	+	+	+																													+					

Note. 2hPG, 2-h postprandial plasma glucose; ACR, urine albumin-creatinine ratio; BMI, body mass index; CVD, cardiovascular disease; CKD, chronic kidney disease; eGFR, estimated glomerular filtration rate; FBG, fasting blood glucose; FPG, fasting plasma glucose; GAD65Ab, glutamic acid decarboxylase-65 antibodies; GADA, glutamic acid decarboxylase antibodies; HbA1c, hemoglobin A1c; HDL-C, HDL-cholesterol; HLA, human leukocyte antigen; HOMA2-IR, homeostasis model assessment 2 of insulin resistance; HOMA2-B, homeostasis model assessment 2 of beta-cell function; HOMA-IR, homeostasis model assessment of insulin resistance; HOMA-B, homeostasis model assessment of beta-cell function; IA-2A, insulinoma-associated antigen-2 autoantibodies; IAA-5.8KD, insulin autoantibody-5.8KD; ICA-40KD, islet cell antibody-40KD; ICA-64KD, islet cell antibody-64KD; ICA-120KD, islet cell antibody- 120KD; ISI, insulin sensitivity index; LDL-C, LDL-cholesterol; mAlb, urine microalbumin; T2D, type 2 diabetes; TC, total cholesterol; TG, triglycerides; TYG, triglycerides/glucose index; UA, uric acid; ZnT8A, zinc transporter 8 autoantibodies.

Table S4. JBI critical appraisal tool for cross-sectional studies.

N	First author, publication year	Q1	Q2	Q3	Q4	Q5	Q6	Q7	Q8	% Yes	Risk of bias
1	Wang et al., 2021 [12]	Y	Y	Y	N	NA	NA	Y	Y	5/6=83.3%	Low
2	Herder et al., 2021 [27]	Y	Y	Y	Y	NA	NA	Y	Y	6/6=100%	Low
3	Song et al., 2022 [14]	Y	Y	Y	Y	NA	NA	Y	Y	6/6=100%	Low
4	Danquah et al., 2023 [37]	N	Y	Y	N	NA	NA	Y	Y	4/6=66.7%	Moderate
5	Huang et al., 2021 [16]	N	Y	Y	N	NA	NA	Y	Y	4/6=66.7%	Moderate
6	Xing et al., 2021 [17]	N	Y	Y	N	NA	NA	Y	Y	4/6=66.7%	Moderate
7	Choi et al., 2022 [34]	N	Y	Y	N	NA	NA	Y	Y	4/6=66.7%	Moderate
8	Li et al., 2023 [22]	Y	Y	Y	N	NA	NA	Y	Y	5/6=83.3%	Low
9	Arora et al., 2024 [31]	Y	Y	Y	Y	NA	NA	Y	Y	6/6=100%	Low
10	Li et al., 2024 [24]	Y	Y	Y	N	NA	NA	Y	Y	5/6=83.3%	Low
11	Bayoumi et al., 2024 [44]	Y	Y	Y	N	NA	NA	Y	Y	5/6=83.3%	Low
12	Cojic et al., 2021 [47]	Y	Y	Y	N	NA	NA	Y	Y	5/6=83.3%	Low
13	Wang et al., 2023 [26]	Y	Y	Y	Y	NA	NA	Y	Y	6/6=100%	Low
14	Cojic et al., 2024 [48]	N	Y	Y	N	NA	NA	Y	Y	4/6=66.7%	Moderate

Note. Y, yes; N, no; NA, not applicable.

Table S5. Risk-of-bias evaluation of included randomized controlled trials using the RoB 2 tool.

N	First author, publication year	Randomization process	Deviations from the intended interventions	Missing outcome data	Measurement of the outcome	Selection of the reported result	Overall risk of bias
1	Pigeyre et al., 2022 [59]	Low risk	Some concerns	Low risk	Low risk	Low risk	Some concerns
2a	Wang et al., 2023 [21]: MARCH	Low risk	Some concerns	Low risk	Low risk	Low risk	Some concerns
2b	Wang et al., 2023 [21]: CONFIDENCE	Low risk	Some concerns	Low risk	Low risk	Low risk	Some concerns
3	Wang et al., 2024 [23]	Low risk	Some concerns	Low risk	Low risk	Low risk	Some concerns
4a	Zou et al., 2022 [58]: CANTATA-M	Low risk	Low risk	Low risk	Low risk	Low risk	Low risk
4b	Zou et al., 2022 [58]: CANTATA-D	Low risk	Low risk	Low risk	Low risk	Low risk	Low risk
4c	Zou et al., 2022 [58]: CANTATA-SU	Low risk	Low risk	Low risk	Low risk	Low risk	Low risk
4d	Zou et al., 2022 [58]: CANVAS	Low risk	Low risk	Low risk	Low risk	Low risk	Low risk
4e	Zou et al., 2022 [58]: CANVAS-R	Low risk	Low risk	Low risk	Low risk	Low risk	Low risk
5a	Abdul-Ghani et al., 2022 [40]: EDICT	Low risk	Some concerns	Low risk	Low risk	Low risk	Some concerns
5b	Abdul-Ghani et al., 2022 [40]: Qatar	Low risk	Some concerns	Low risk	Low risk	Low risk	Some concerns

Table S6. Evaluation of quality of cohort studies using the NHLBI tool.

N	First author, publication year	Q1	Q2	Q3	Q4	Q5	Q6	Q7	Q8	Q9	Q10	Q11	Q12	Q13	Q14	Total score
1	Xiong et al., 2021 [13]	Y	N	Y	Y	NA	Y	Y	Y	Y	N	Y	NA	Y	NA	9/11=81.8%
2	Zhang et al., 2022 [15]	Y	N	Y	Y	NA	Y	Y	Y	Y	N	Y	NA	NR	NA	8/10=80%
3	Tanabe et al., 2022 [28]	Y	N	Y	Y	NA	Y	Y	Y	Y	N	Y	NA	NR	NA	8/10=80%
4	Saito et al., 2022 [29]	Y	N	Y	Y	NA	Y	Y	Y	Y	N	Y	NA	N	NA	8/11=72.7%
5	Lu et al., 2024 [33]	Y	Y	Y	Y	NA	Y	Y	Y	Y	N	Y	NA	NR	NA	9/10=90%
6	Anjana et al., 2020 [30]	Y	Y	Y	Y	NA	Y	Y	Y	Y	N	Y	NA	NR	NA	9/10=90%
7	Gao et al., 2022 [18]	Y	N	Y	Y	NA	Y	Y	Y	Y	N	Y	NA	NR	NA	8/10=80%
8	Christensen et al., 2022 [38]	Y	Y	Y	Y	NA	Y	Y	Y	Y	N	Y	NA	NR	NA	9/10=90%
9	Peng et al., 2022 [19]	Y	N	Y	Y	NA	Y	Y	Y	Y	N	Y	NA	NA	NA	8/10=80%
10	Wang et al., 2022 [39]	Y	N	Y	Y	NA	Y	Y	Y	Y	N	Y	NA	NR	NA	8/10=80%
11	Preechasuk et al., 2022 [41]	Y	N	Y	Y	NA	Y	Y	Y	Y	N	Y	NA	NR	NA	8/10=80%
12	Wang et al., 2022 [20]	Y	N	Y	Y	NA	Y	Y	Y	Y	N	Y	NA	N	NA	8/11=72.7%
13	Li et al., 2023 [42]	Y	Y	Y	Y	NA	Y	Y	Y	Y	N	Y	NA	Y	NA	10/11=90.9%
14	Hwang et al., 2023 [43]	Y	N	Y	Y	NA	Y	Y	Y	Y	Y	Y	NA	NR	NA	9/10=90%
15	Tripathi et al., 2024 [32]	Y	N	Y	Y	NA	Y	Y	Y	Y	N	Y	NA	NR	NA	8/10=80%
16	Li et al., 2024 [45]	Y	Y	Y	Y	NA	Y	Y	Y	Y	Y	Y	NA	Y	NA	11/11=100%
17	Lugner et al., 2021 [46]	Y	N	Y	Y	NA	Y	Y	Y	Y	N	Y	NA	NR	NA	8/10=80%
18	Grimsmann et al., 2022 [49]	Y	Y	Y	Y	NA	Y	Y	Y	Y	N	Y	NA	N	NA	9/11=81.8%
19	Manzini et al., 2022 [50]	Y	N	Y	Y	NA	Y	Y	Y	Y	N	Y	NA	Y	NA	9/11=81.8%
20	Wang et al., 2023 [25]	Y	N	Y	Y	NA	Y	Y	Y	Y	N	Y	NA	NA	NA	8/10=80%
21	Abbasi et al., 2023 [35]	Y	Y	Y	Y	NA	Y	Y	Y	Y	N	Y	NA	NR	NA	9/10=90%
22	Somolinos-Simon et al., 2024 [36]	Y	N	Y	Y	NA	Y	Y	Y	Y	Y	Y	NA	N	NA	9/11=81.8%

Note. Y, yes; N, no; NA, not applicable; NR, not reported.

Table S7. Characteristics of clusters aligned with a previously described diabetes classification (SAID, SIDD, SIRD, MOD, MARD).

N	First author, publication year	Cluster (%)	Age at onset of DM or at atudy enrollment	BMI	Insulin secretion	Insulin Resistance	Glycemic control	Auto-antibodies
1	Wang et al., 2021 [12]	SAID (4.4%)	-	Normal	Severe deficiency *	Mild resistance	Very poor	+
		SIDD (20.5%)	Younger age	Normal	Severe deficiency	Severe resistance	Very poor	
		SIRD (19%)	-	Obesity I	Moderate deficiency	Severe resistance *	Poor	
		MOD (34.6%)	-	Normal	Moderate/Mild deficiency	Moderate/Severe resistance	Suboptimal	
		MARD (21.6%)	Older age	Normal	Moderate deficiency	Severe resistance	Very poor *	
2	Herder et al., 2021 [27]	SAID (21%)	Younger age	Normal	Moderate deficiency	No resistance	Good	+
		SIDD (3%)	-	Overweight	Moderate deficiency *	No resistance	Poor	
		SIRD (9%)	-	Obesity I *	Increased secretion	Severe resistance *	Good	
		MOD (32%)	-	Obesity I	Normal secretion	Severe resistance	Good	
		MARD (35%)	Older age	Overweight	Normal secretion	No resistance	Good	
3	Xiong et al., 2021 [13]:	SIRD (1.9%)	-	Normal	Moderate deficiency	Severe resistance	Very poor	
		SIDD (19.9%))	-	Normal	Severe deficiency	No resistance	Very poor *	
		MOD (17.1%)	Younger age	Overweight *	Moderate deficiency	No resistance	Very poor	
		SAID (2.6%)	-	Normal	Moderate deficiency	No resistance	Poor	+
		UARD (12.3%)	-	Overweight	Increased secretion	Mild resistance	Suboptimal	
		MARD (24.7%)	Older age	Normal	Mild deficiency	No resistance	Suboptimal	
		IRD (21.5%)	-	Normal	Moderate deficiency	No resistance	Poor	
4	Song et al., 2022 [14]	SAID (4.1%)	Younger age	Normal	Severe deficiency *	Moderate/Severe resistance	Very poor *	+
		SIDD (44.4%)	-	Normal	Severe deficiency	Severe resistance	Very poor	
		SIRD (7.9%)	-	Overweight/Obesity I	Normal secretion/Mild deficiency	Severe resistance *	Poor/Very poor	
		MOD (20.4%)	-	Obesity I	Moderate/Severe deficiency	Severe resistance	Very poor	
		MARD (23.2%)	Older age	Normal/Overweight	Mild/Moderate deficiency	Severe resistance	Suboptimal	
5	Zhang et al., 2022 [15]	SAID (3.8%)	-	Normal	Moderate deficiency	No resistance	Very poor	+
		SIDD (27.6%)	-	Normal	Severe deficiency	No resistance	Very poor *	
		SIRD (17.2%)	Younger age	Overweight *	Moderate deficiency	Severe resistance	Very poor	
		MOD (21.3%)	-	Overweight	Increased secretion	Mild resistance	Good	
		MARD (30%)	Older age	Normal	Mild deficiency	No resistance	Suboptimal	
6		MARD (38%)	Older age	Overweight	Normal secretion	-	Good	

	Pigeyre et al., 2022 [59]	MOD (23%)	Younger age	Obesity I *	Normal secretion	-	Good	
		SIDD (23%)	-	Overweight	Normal secretion	-	Suboptimal	
		SIRD (13%)	-	Obesity I	Increased secretion	-	Good	
		SAID (3%)	-	Overweight	Normal secretion	-	Good	+
7	Tanabe et al., 2022 [28]	SAID (10.2%)	Younger age	Normal	Severe deficiency	No resistance	Suboptimal	+
		SIDD (15.4%)	-	Overweight	Moderate deficiency	No resistance	Very poor	
		SIRD (13.3%)	-	Obesity I	Increased secretion	Severe resistance	Good	
		MOD (25.4%)	-	Overweight	Normal secretion/Mild deficiency	Mild resistance	Good	
		MARD (35.7%)	Older age	Normal	Mild deficiency	No resistance	Good	
8	Saito et al., 2022 [29]	SAID (16%)	-	Normal	Severe deficiency	No resistance	Very poor	+
		SIDD (18%)	-	Normal	Severe deficiency *	No resistance	Very poor *	
		SIRD (14%)	-	Overweight	Normal secretion	Severe resistance	Very poor	
		MOD (18%)	Younger age	Overweight *	Moderate deficiency	Mild resistance	Very poor	
		MARD (33%)	Older age	Normal	Moderate deficiency	No resistance	Very poor	
9	Danquah et al., 2023 [37]	Obesity-related (73%)	Older age	Overweight	Moderate deficiency	No resistance	Suboptimal	
		Age-related (10%)	-	Overweight	Moderate deficiency	Mild resistance	Suboptimal	
		Autoimmune-related (5%)	-	Obesity I	Severe deficiency *	Mild resistance	Poor	+
		Insulin-deficient (7%)	Younger age	Overweight	Severe deficiency	Mild resistance	Suboptimal	
		Insulin-resistant (5%)	-	Overweight	Increased secretion	Severe resistance	Good	
10	Lu et al., 2024 [33]	SAID (9%)	-	Obesity I	Mild deficiency	Severe resistance	Poor	+
		SIDD (24%)	Younger age	Obesity I	Moderate deficiency	Severe resistance *	Very poor	
		SIRD (14%)	-	Obesity I	Increased secretion	Severe resistance	Suboptimal	
		MOD (32%)	-	Obesity II	Mild deficiency	Severe resistance	Suboptimal	
		MARD (21%)	Older age	Overweight	Mild deficiency	Moderate resistance	Suboptimal	
11	Anjana et al., 2020 [30]	SIDD (26.2%)	-	Normal	Moderate deficiency	Mild resistance	Very poor *	
		IROD (25.9%)	-	Obesity I	Increased secretion	Severe resistance	Poor	
		CIRDD (12.1%)	Younger age	Overweight	Mild deficiency	Moderate resistance	Very poor	
		MARD (35.8%)	Older age	Overweight	Normal secretion	Mild resistance	Suboptimal	
12	Huang et al., 2021 [16]	MOD (19.1%)	-	Overweight	Increased secretion	Severe resistance	Poor	
		SIDD (25.6%)	Younger age	Normal	Normal secretion	Severe resistance	Very poor	
		SIRD (8.9%)	-	Normal	Increased secretion *	Severe resistance *	Suboptimal	
		MARD (46.3%)	Older age	Normal	Increased secretion	Severe resistance	Poor	
13	Xing et al., 2021 [17]	SIDD (21%)	Older age	Normal	Moderate/Severe deficiency	No resistance	Very poor	
		SIRD (21%)	-	Overweight	Increased secretion	Mild resistance	Normal/Suboptimal	

		MOD (25%)	Younger age	Overweight *	Moderate deficiency	No resistance/Mild resistance	Poor/Very poor	
		MARD (33%)	-	Normal	Mild deficiency	No resistance	Suboptimal	
14	Gao et al., 2022 [18]	SIRD (10.2%)	-	Overweight	Increased secretion *	Severe resistance *	Good	
		SIDD (12.9%)	-	Normal	Moderate deficiency	Severe resistance	Very poor	
		MOD (30%)	Younger age	Overweight *	Increased secretion	Severe resistance	Good	
		MARD (46.9%)	Older age	Normal	Normal secretion	Severe resistance	Good	
15	Christensen et al., 2022 [38]: Replication of the Swedish T2D clusters	SIDD (8%)	-	Obesity I	Mild deficiency	Severe resistance	Very poor	
		SIRD (23%)	-	Obesity II	Increased secretion	Severe resistance *	Good	
		MOD (26%)	Younger age	Obesity I	Normal secretion	Severe resistance	Good	
		MARD (42%)	Older age	Overweight	Normal secretion	Moderate resistance	Good	
	Christensen et al., 2022 [38]: New DD2 clusters	New Cluster 1 (12%)	Younger age	Obesity I	Mild deficiency	Severe resistance	Very poor	
		New Cluster 2 (32%)	-	Obesity II *	Increased secretion	Severe resistance	Good	
		New Cluster 3 (56%)	Older age	Overweight	Moderate deficiency	Moderate resistance	Good	
16	Peng et al., 2022 [19]	MOD (17.5%)	-	Obesity I	Increased secretion	Severe resistance	Poor	
		SIDD (24.8%)	Younger age	Normal	Normal secretion	Severe resistance	Very poor	
		SIRD (16.8%)	-	Normal	Increased secretion *	Severe resistance *	Suboptimal	
		MARD (40.9%)	Older age	Normal	Increased secretion	Severe resistance	Suboptimal	
17	Zou et al., 2022 [58]	MARD (40.3%)	Older age	Overweight	Moderate deficiency	No resistance	Suboptimal	
		MOD (22.8%)	Younger age	Obesity II	Moderate deficiency	Mild resistance	Suboptimal	
		SIDD (20.5%)	-	Obesity I	Severe deficiency	No resistance	Very poor	
		SIRD (16.4%)	-	Obesity I	Normal secretion	Severe resistance *	Suboptimal	
18	Choi et al., 2022 [34]	MARD (32.6%)	Older age	Obesity I	Mild deficiency	Moderate/Severe resistance	Good	
		SIDD (33.7%)	Younger age	Obesity I	Severe deficiency	Moderate resistance	Suboptimal	
		MOD (20%)	-	Obesity I / II	Normal secretion	Severe resistance	Good	
		SIRD (13.7%))	-	Obesity II	Increased secretion	Severe resistance *	Good	
19	Wang et al., 2022 [39]	MOD (45%)	-	Obesity I	Normal secretion	Mild resistance	Good	
		SIRD-RII (19%)	Younger age	Obesity I *	Moderate deficiency	Severe resistance	Very poor	
		MARD-II (36%)	Older age	Normal	Mild deficiency	No resistance	Suboptimal	
20	Abdul-Ghani et al., 2022 [40]: EDICT study	Group 1 (47%)	Younger age	Obesity II	Severe deficiency	Severe resistance	Very poor	
		Group 2 (18%)	-	Obesity III	Increased secretion	Severe resistance *	Poor	
		Group 3 (35%)	Older age	Obesity I	Mild deficiency	Severe resistance	Suboptimal	
	Abdul-Ghani et al., 2022 [40]: Qatar study	Group 1 (38%)	Younger age	Overweight	Severe deficiency *	Severe resistance	Very poor *	
		Group 2 18%)	-	Obesity II	Mild deficiency	Severe resistance *	Very poor	
		Group 3 (44%)	Older age	Overweight	Severe deficiency	Severe resistance	Very poor	

21	Preechasuk et al., 2022 [41]	SIDD (18.6%)	-	Normal	-	-	Very poor *	
		MSD (11.8%)	-	Overweight	-	-	Very poor	
		MOD (23.3%))	Younger age	Obesity I	-	-	Poor	
		MARD (46.3%)	Older age	Overweight	-	-	Suboptimal	
22	Wang et al., 2022 [20]	MARD (33.7%)	Older age	Normal	Mild deficiency	No resistance	Good	
		MOD (40.4%)	Younger age	Overweight	Mild deficiency	Mild resistance	Good	
		SIDRD (7.7%)	-	Overweight	Severe deficiency	Severe resistance	Very poor	
		SOIRD (18.2%)	-	Overweight *	Increased secretion	Severe resistance *	Good	
23	Wang et al., 2023 [21]: MARCH cohort	MARD (35.1%)	Older age	Normal	Moderate deficiency	Severe resistance	Suboptimal	
		MOD (34.4%))	Younger age	Overweight *	Mild defficiency	Severe resistance	Suboptimal	
		SIDD (19.2%)	-	Overweight	Moderate deficiency *	Severe resistance	Very poor	
		SIRD (11.4%)	-	Overweight	Increased secretion	Severe resistance *	Good	
	Wang et al., 2023 [21]: CONFIDENCE cohort	MARD	-	Normal	Moderate deficiency	Severe resistance	Suboptimal	
		MOD	Younger age	Overweight *	Mild defficiency	Severe resistance	Poor	
		SIDD	-	Normal	Moderate deficiency *	Severe resistance	Very poor	
		SIRD	Older age	Overweight	Increased secretion	Severe resistance *	Suboptimal	
24	Li et al., 2023 [42]	RHAP-SIDD (12.4%)	-	Overweight	Increased secretion	-	Suboptimal	
		RHAP-SIRD (21.7%)	Older age	Overweight	Increased secretion *	-	Good	
		RHAP-MOD (17.7%)	Younger age	Obesity II	Increased secretion *	-	Good	
		RHAP-MD (29.3%)	-	Overweight	Increased secretion	-	Good	
		RHAP-MDH (18.8%)	-	Overweight	Increased secretion	-	Good	
25	Hwang et al., 2023 [43]	SIRD (22.4%)	-	Overweight *	Increased secretion	Mild resistance	Good	
		MARD (32.7%)	Older age	Normal	Normal secretion	No resistance	Good	
		MOD (32.7%)	Younger age	Overweight	Mild deficiency	No resistance	Good	
		SIDD (12.3%)	-	Overweight	Moderate deficiency	No resistance	Very poor	
26	Li et al., 2023 [22]	MARD (30.7%)	Younger age	Normal	Normal secretion	-	Poor	
		SIDD (25.8%)	-	Normal	Normal secretion	-	Very poor *	
		MOD (24.9%)	Older age	Overweight *	Increased secretion *	-	Suboptimal	
		SIRD (18.7%)	-	Overweight	Increased secretion	-	Very poor	
27	Wang et al., 2024 [23]	MARD (34.1%)	Older age	Normal	Moderate deficiency	Severe resistance	Good	
		MOD (34.1%)	Younger age	Overweight *	Mild deficiency	Severe resistance	Suboptimal	
		SIDD (20.3%)	-	Overweight	Moderate deficiency *	Severe resistance	Very poor	
		SIRD (11.5%)	-	Overweight	Increased secretion	Severe resistance *	Good	
28	Arora et al., 2024 [31]	SIDD (15%)	-	Normal	Moderate deficiency *	Mild resistance	Very poor *	

		SIRD (22%)	-	Obesity I	Normal secretion	Moderate resistance *	Poor	
		MOD (35%)	Younger age	Overweight	Moderate deficiency	Mild resistance	Very poor	
		MARD (27%)	Older age	Overweight	Moderate deficiency	Moderate resistance	Very poor	
29	Li et al., 2024 [24]	SOIRD (32.4%)	Younger age	Overweight *	Normal secretion	Moderate resistance *	Good	
		SIDD (10.3%)	-	Overweight	Severe deficiency	Moderate	Very poor	
		MARD (27.8%)	Older age	Overweight	Mild deficiency	Mild resistance	Good	
		MIDD (29.5%)	-	Normal	Moderate deficiency	No resistance	Good	
30	Tripathi et al., 2024 [32]	SIDD (34.5%)	Younger age	Normal	Moderate deficiency	No resistance	Very poor	
		SIRD (13.5%)	Older age	Obesity I	Increased secretion	Severe resistance	Good	
		MOD (52%)	-	Overweight	Normal secretion	Mild resistance	Good	
31	Bayoumi et al., 2024 [44]	SIRD (8%)	-	Obesity I	Increased secretion *	Severe resistance *	Suboptimal	
		SIDD (16%)	-	Obesity I	Moderate deficiency	Severe resistance	Very poor	
		MARD (25%)	Older age	Overweight	Increased secretion	Severe resistance	Good	
		MOD (21%)	-	Obesity II	Normal secretion	Severe resistance	Suboptimal	
		MEOD (30%)	Younger age	Overweight	Mild deficiency	Mild resistance	Suboptimal	
32	Li et al., 2024 [45]: DCS cohort	RHAP-SIDD (11.9%)	-	Overweight	Increased secretion	-	Very poor	
		RHAP-SIRD (21.5%)	Older age	Obesity I	Increased secretion *	-	Good	
		RHAP-MOD (16.4%)	Younger age	Obesity II	Increased secretion	-	Suboptimal	
		RHAP-MD (29.6%)	-	Overweight	Increased secretion	-	Good	
		RHAP-MDH (20.7%)	-	Overweight	Increased secretion	-	Good	
	Li et al., 2024 [45]: GoDARTS cohort	RHAP-SIDD (17.8%)	-	Overweight	Increased secretion	-	Very poor	
		RHAP-SIRD (17.3%)	-	Obesity I	Increased secretion *	-	Suboptimal	
		RHAP-MOD (18.6%)	Younger age	Obesity III *	Increased secretion	-	Suboptimal	
		RHAP-MD (28%)	-	Obesity I	Increased secretion	-	Good	
		RHAP-MDH (18.3%)	Older age	Overweight	Increased secretion	-	Suboptimal	

BMI, body mass index; CIRDD, combined insulin-resistant and deficient diabetes; DM, diabetes mellitus; IRD, inheritance-related diabetes; IROD, insulin-resistant obese diabetes; MARD, mild age-related diabetes; MARD-II, mild age-related diabetes with insulin insufficiency; MD, mild diabetes; MDH, mild diabetes with high HDL-cholesterol; MEOD, mild early onset diabetes; MIDD, mild insulin-deficient diabetes; MOD, mild obesity-related diabetes; MSD, metabolic syndrome diabetes; SAID, severe autoimmune diabetes; SIDD, severe insulin-deficient diabetes; SIDRD, severe insulin-deficient and insulin-resistant diabetes; SIRD, severe insulin-resistant diabetes; SIRD-RII, severe insulin-resistant diabetes with relative insulin insufficiency; SOIRD, severe obesity-related and insulin-resistant diabetes; UARD, uric acid-related diabetes.

Insulin resistance was assessed using HOMA-IR and HOMA2-IR. For HOMA-IR [16, 20, 24, 30, 31, 34, 37, 40, 44], thresholds were: no resistance (≤2.5), mild insulin resistance (>2.5–3.0), moderate insulin resistance (>3.0–4.0), and severe insulin resistance (>4.0). For HOMA2-IR [12-15, 17-19, 21, 23, 27-29, 32, 33, 38, 39, 43, 58], thresholds were: no resistance (≤1.8), mild insulin resistance (>1.8–2.2), moderate insulin resistance (>2.2–2.5), and severe insulin resistance (>2.5).

Insulin secretion was assessed using HOMA2-B, HOMA-B, and fasting C-peptide levels. For HOMA2-B and HOMA-B [12-21, 23, 24, 27-34, 37-40, 43, 44, 58], thresholds were: normal secretion (70–100%), mild deficiency (50–69%), moderate deficiency (30–49%), severe deficiency (<30%), and increased secretion (>100%). Fasting C-peptide [22, 42, 45, 59] thresholds were: normal secretion (0.78–1.89 ng/mL [0.26–0.62 nmol/L]), mild deficiency (0.47–0.77 ng/mL [0.16–0.25 nmol/L]), moderate deficiency (0.23–0.46 ng/mL [0.08–0.15 nmol/L]), severe deficiency (<0.23 ng/mL [<0.08 nmol/L]), and increased secretion (>1.89 ng/mL [>0.62 nmol/L]).

Glycemic control was defined by HbA1c treatment categories: good (<7.0%), suboptimal (7.0–7.9%), poor (8.0–8.9%), and very poor (≥9.0%).

BMI was categorized as follows: normal weight (18.5–24.9 kg/m²), overweight (25.0–29.9 kg/m²), obesity class I (30.0–34.9 kg/m²), obesity class II (35.0–39.9 kg/m²), and obesity class III (≥40.0 kg/m²).

Insulin secretion: * indicates the highest or lowest value only when multiple clusters fall within the same category.
Insulin resistance: * indicates worse insulin resistance when multiple clusters fall within the same category.
BMI: * indicates the highest BMI among clusters within the same category.
Glycemic control: * indicates the poorest glycemic control among clusters within the same category.
* not used when differences are evident from category labels.

Table S8. Characteristics of clusters identified using alternative clustering methods.

N	First author, publication year	Cluster (%)	Study poplulation	Age at onset of DM or at atudy enrollment	BMI	Cardiometabolic profile	Diabetes complications and comorbidities
1	Lugner et al., 2021 [46]	Cluster 1 (10.6%)	T2D	-	Obesity I	Less favorable lipid profile, very poor glycemic control	-
		Cluster 2 (24.4%)	T2D	-	Obesity I	Higher BP levels, good glycemic control	-
		Cluster 3 (36.3%)	T2D	Older age	Overweight	More favorable lipid profile, good glycemic control	-
		Cluster 4 (28.7%)	T2D	Younger age	Obesity I *	Good glycemic control	-
2	Cojic et al., 2021 [47]	Cluster 1 (18.9%)	T2D	Older age	Overweight	Increased insulin secretion, severe IR, suboptimal glycemic control	-
		Cluster 2 (24.2%)	T2D	-	Overweight	Normal insulin secretion, mild IR, good glycemic control	-
		Cluster 3 (27.4%)	T2D	Younger age	Obesity I	Increased insulin secretion, moderate IR, better lipid profile, good glycemic control	-
		Cluster 4 (29.5%)	T2D	-	Overweight	Increased insulin secretion *, severe IR *, worse lipid profile; higher BP, good glycemic control	-
3	Grimsmann et al., 2022 [49]	Cluster 1 (12%)	31% T1D/69% T2D	Younger age	Overweight	Suboptimal glycemic control	Reference group (complication comparison)
		Cluster 2 (12%)	45% T1D/55% T2D	-	Normal	Lower percentage of individuals having ≥3 components of the metabolic syndrome, very poor glycemic control *	Highest risk of DR, increased risk of CKD, and neuropathy (vs. Cluster 1)
		Cluster 3 (11%):	12% T1D/88% T2D	-	Obesity I	Very poor glycemic control	Highest risk of CKD, CVD, neuropathy, and increased risk of DR (vs. Cluster 1)
		Cluster 4 (15%)	2% T1D/98% T2D	-	Obesity I	Good glycemic control	Reduced risk of CKD, increased risk of neuropathy (vs. Cluster 1)
		Cluster 5 (13%)	10% T1D/90% T2D	-	Overweight	Good glycemic control	Lowest risk of CKD, increased risk of CVD, neuropathy (vs. Cluster 1)
		Cluster 6 (15%)	4% T1D/96% T2D	-	Overweight	Very poor glycemic control	Increased risk of CKD, CVD, DR, neuropathy (vs. Cluster 1)
		Cluster 7 (6%)	2% T1D/98% T2D	-	Obesity III	Higher percentage of individuals having ≥3 components of the metabolic syndrome, suboptimal glycemic control	Highest risk of CVD, elevated risk of CKD and neuropathy (vs. Cluster 1)
		Cluster 8 (16%)	2% T1D/98% T2D	Older age	Overweight	Good glycemic control	Increased risk of CVD (vs. Cluster 1)
4		Neuropathic cluster (7.6%)	T2D	-	Obesity I	Suboptimal glycemic control	Highest percentage of neuropathy

	Manzini et al., 2022 [50]	Hypercholesteraemic cluster (13.3%)	T2D	Younger age	Overweight	More hypercholesteraemic lipid profile, suboptimal glycemic control	-
		Multiple complications cluster (5.1%)	T2D	-	Obesity I *	More hypertriglyceridaemic lipid profile, higher BP, suboptimal glycemic control	Highest percentage of renal complications
		Vascular disease cluster (11.8%)	T2D	Older age	Overweight	Suboptimal glycemic control	Highest percentage of CVD
		Hypertensive cluster (37.5%)	T2D	-	Obesity I	Suboptimal glycemic control	Highest percentage of hypertension
		Retinopathy cluster (11.2%)	T2D	-	Obesity I	Suboptimal glycemic control	Highest percentage of ophthalmological complications
		Metabolic cluster (13.5%)	T2D	-	Obesity I	Suboptimal glycemic control *	-
5	Wang et al., 2023 [25]	Low-Risk (49.1%)	T2D	-	Normal	Normal insulin secretion, moderate IR, more favorable lipid profile, suboptimal glycemic control	Reference group (complication comparison)
		Medium-Low-Risk (37.9%)	T2D	Older age	Overweight	Increased insulin secretion, severe IR, higher BP levels, very poor glycemic control	Increased risk of microangiopathy, diabetic nephropathy, and DR (vs. Low-Risk)
		Medium-Risk (5.9%)	T2D	-	Overweight	Increased insulin secretion *, severe IR *, very poor glycemic control *	Increased risk of microangiopathy, diabetic nephropathy, and DR, reduced risk of ASCVD risk (vs. Low-Risk)
		High-Risk (7.2%)	T2D	Younger age	Overweight *	Increased insulin secretion, severe IR, less favorable lipid profile, very poor glycemic control	Highest risk of microangiopathy, diabetic nephropathy, and DR (vs. Low-Risk)
6	Abbasi et al., 2023 [35]. Pediatric population	Cluster 1 (22.7%)	T2D, pediatric cohort	-	-	More favorable lipid profile, very poor glycemic control *	Highest prevalence of DKA
		Cluster 2 (10.7%)	T2D, pediatric cohort	-	-	Less favorable lipid profile, very poor glycemic control	Highest prevalence of PCOS, microalbuminuria within 1 year, dyslipidemia
		Cluster 3 (29.2%)	T2D, pediatric cohort	Younger age	-	Suboptimal glycemic control	-
		Cluster 4 (10.9%)	T2D, pediatric cohort	-	-	Very poor glycemic control	-
		Cluster 5 (26.5%)	T2D, pediatric cohort	Older age	-	Poor glycemic control	-
7	Wang et al., 2023 [26]	Cluster 1 (9.9%)	T1D, mixed-age cohort	Older age	Normal	Moderate deficiency, very poor glycemic control	-
		Cluster 2 (6.1%)	T1D, mixed-age cohort	Younger age	Normal	Severe deficiency, very poor glycemic control	Highest rates of DKA and DK
		Cluster 3 (13.7%)	T1D, mixed-age cohort	-	Normal	Mild deficiency, poor glycemic control	-
		Cluster 4 (30.7%)	T1D, mixed-age cohort	-	Normal	Moderate deficiency, very poor glycemic control *	Lowest rates of DKA and DK
		Cluster 5 (39.6%)	T1D, mixed-age cohort	-	Close to the underweight cutoff	Moderate deficiency, very poor glycemic control	-
8	Cojic et al., 2024 [48]	Cluster 1 (11.3%)	T2D	-	Overweight *	Less favorable lipid profile, poor glycemic control	-

		Cluster 2 (49.8%):	T2D	-	Overweight	Suboptimal glycemic control	Higher prevalence of CAD (vs. Cluster 4), neuropathy (vs. Cluster 1)
		Cluster 3 (17.2%)	T2D	Older age	Overweight	More favorable lipid profile, suboptimal glycemic control	Higher prevalence of CAD (vs. Cluster 4), nephropathy, hypertension (vs. all other clusters)
		Cluster 4 (21.7%)	T2D	Younger age	Overweight	Good glycemic control	-
9	Somolinos-Simon et al., 2024 [36]	Cluster 1 (31.7%)	T1D	-	Normal	Suboptimal glycemic control	Reference group (complication comparison)
		Cluster 2 (22.6%)	T1D	-	Overweight	HDL-favorable lipid profile, suboptimal glycemic control	Highest risk of DR, CAD, and autonomic neuropathy, and increased risk of albuminuria (vs. Cluster 1)
		Cluster 3 (13.3%)	T1D	Younger age	Overweight	Unfavorable lipid profile, very poor glycemic control	Greatest risk of albuminuria, increased risk of depression and DKA (vs. Cluster 1)
		Cluster 4 (15.3%)	T1D	-	Obesity II	Unfavorable lipid profile, suboptimal glycemic control	Increased risk of DR, albuminuria, autonomic neuropathy, hypertension, and severe hypoglycemia (vs. Cluster 1)
		Cluster 5 (17.1%)	T1D	Older age	Overweight	HDL-favorable lipid profile, suboptimal glycemic control	Elevated risk of CAD, highest risk of hypertension and severe hypoglycemia, lowest risk of DR (vs. Cluster 1)

ASCVD, atherosclerotic cardiovascular disease; BMI, body mass index; BP, blood pressure; CAD, coronary artery disease; CKD, chronic kidney disease; CVD, cardiovascular disease; DK, diabetic ketosis; DKA, diabetic ketoacidosis; DM, diabetes mellitus; DR, diabetic retinopathy, HDL-C, HDL-cholesterol; IR, insulin resistance; T1D, type 1 diabetes mellitus; T2D, type 2 diabetes mellitus.

Insulin resistance was assessed using HOMA-IR with the following thresholds: no resistance (≤2.5), mild insulin resistance (>2.5–3.0), moderate insulin resistance (>3.0–4.0), and severe insulin resistance (>4.0).

Insulin secretion was assessed using fasting C-peptide and insulin levels. For fasting C-peptide [26], thresholds were: normal secretion (0.78–1.89 ng/mL [258–625 pmol/L]), mild deficiency (0.47–0.77 ng/mL [156–255 pmol/L]), moderate deficiency (0.23–0.46 ng/mL [76–152 pmol/L]), severe deficiency (<0.23 ng/mL [<76 pmol/L]), and increased secretion (>1.89 ng/mL [>625 pmol/L]). For fasting insulin [25, 47], thresholds were: normal secretion (6.1–10.0 µU/mL), mild deficiency (4.1–6.0 µU/mL), moderate deficiency (2.0–4.0 µU/mL), severe deficiency (<2.0 µU/mL), and increased secretion (>10.0–15.0 µU/mL).

Glycemic control was defined by HbA1c treatment categories: good (<7.0%), suboptimal (7.0–7.9%), poor (8.0–8.9%), and very poor (≥9.0%).

BMI was categorized as follows: normal weight (18.5–24.9 kg/m^2), overweight (25.0–29.9 kg/m^2), obesity class I (30.0–34.9 kg/m^2), obesity class II (35.0–39.9 kg/m^2), and obesity class III (≥40.0 kg/m^2). BMI categories for children and adolescents were defined according to CDC BMI-for-age percentiles: underweight (<5th percentile), healthy weight (5th–<85th percentile), overweight (85th–<95th percentile), and obese (≥95th percentile).

Insulin secretion: * indicates the highest or lowest value only when multiple clusters fall within the same category.
Insulin resistance: * indicates worse insulin resistance when multiple clusters fall within the same category.
BMI: * indicates the highest BMI among clusters within the same category.
Glycemic control: * indicates the poorest glycemic control among clusters within the same category.
* not used when differences are evident from category labels.
